# Recording development with single cell dynamic lineage tracing

**DOI:** 10.1242/dev.169730

**Published:** 2019-06-27

**Authors:** Aaron McKenna, James A. Gagnon

**Affiliations:** 1 Department of Molecular and Systems Biology, Geisel School of Medicine, Dartmouth College, Hanover, NH 03755, USA; 2 Center for Cell and Genome Science, University of Utah, Salt Lake City, UT 84112, USA; 3 School of Biological Sciences, University of Utah, Salt Lake City, UT 84112, USA

**Keywords:** Lineage tracing, Lineage trees, Single cell

## Abstract

Every animal grows from a single fertilized egg into an intricate network of cell types and organ systems. This process is captured in a lineage tree: a diagram of every cell's ancestry back to the founding zygote. Biologists have long sought to trace this cell lineage tree in individual organisms and have developed a variety of technologies to map the progeny of specific cells. However, there are billions to trillions of cells in complex organisms, and conventional approaches can only map a limited number of clonal populations per experiment. A new generation of tools that use molecular recording methods integrated with single cell profiling technologies may provide a solution. Here, we summarize recent breakthroughs in these technologies, outline experimental and computational challenges, and discuss biological questions that can be addressed using single cell dynamic lineage tracing.

## Abstract (precis)

**Summary:** A review of recent breakthroughs in single cell dynamic lineage tracing, outlining experimental and computational challenges and discussing applications for developmental biology.

## Introduction

**Fig. 1.**
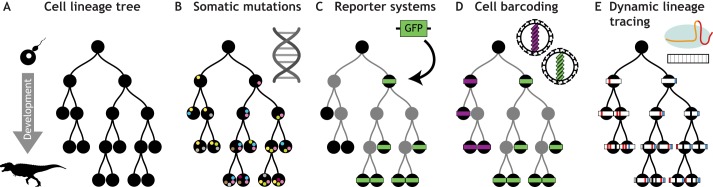
**Lineage tracing in development.** (A) The cell lineage tree describes the successive cell divisions that generate all of the organs and tissues within an organism. Lineage tracing aims to capture these cellular relationships. (B) Rare mutations (marked by colored dots) occur sporadically across the genome during development. These mutations can be used to trace complete lineage trees, but require whole- or partial-genome sequencing. (C) A classic labeling approach is to tag a subset of cells with a dye or fluorescent marker such as GFP (green); this approach is good for tagging all descendants of the marked cell but does not reveal relationships within the marked population. (D) Cellular barcoding, using viruses or transposons, can label a population of cells with unique identifiers (purple and green). Descendant cells can then be assigned to common progenitors. These approaches can capture clonal relationships but cannot infer relationships within each subpopulation. (E) Dynamic lineage-tracing approaches add increasing information over developmental time. These approaches can assign cells to progenitors and determine branches within the lineage tree.

The development of plants and animals has long been a source of fascination to those interested in the construction of complex organisms. In particular, researchers have sought to understand the collective pattern of cell divisions – termed a ‘cell lineage tree’ (Fig. 1A) – that transforms a single-celled zygote into a fully-formed adult organism. Two strategies to map cell lineage trees have emerged: imaging-based approaches and genetic approaches. Advances in imaging-based lineage tracing have been driven by technological progress in microscopy throughout the 20th century. Such advances include the work of Nicole Le Douarin, who generated quail/chicken chimeras, and the dye labeling and transplantation experiments carried out in frogs and fish by Spemann and others (Kretzschmar and Watt, 2012). The culmination was the generation of the complete *C. elegans* lineage tree by Sir John Sulston and colleagues (Sulston et al., 1983). More recently, advances in fluorescent protein engineering, and in confocal and light sheet microscopy, have enabled the lineage tracing of single cells within intact, living animals. This branch of lineage tracing has been recently reviewed (Kretzschmar and Watt, 2012; Liu and Keller, 2016). Suffice to say, these tools have advanced significantly in recent years, recording development through time with incredible cellular, and even subcellular, resolution (Liu et al., 2018; McDole et al., 2018). These methods all record lineages in living animals or tissues, and retain the spatial context that is so essential for understanding the growth and maintenance of animals. However, imaging-based approaches are limited with regard to their temporal and molecular resolution. For example, animal development often occurs over timescales that make imaging impractical, and physiological growth conditions may be impossible to recreate under a microscope. It also remains difficult to capture detailed molecular information, such as the transcriptional state of individual cells, in conjunction with imaging-based lineage measurements, although recent advances in the scale of *in situ* imaging techniques are rapidly upending this assumption (Codeluppi et al., 2018 preprint; Shah et al., 2018; Wang et al., 2018; Rodriques et al., 2019; Eng et al., 2019).

Genetic or epigenetic changes can be used as an alternative to imaging-based techniques to infer lineage. A number of research groups have leveraged these naturally occurring changes to trace lineages, both in development and in cancer. Pioneering work in the 1960s, for example, used the silencing of specific alleles by X-chromosome inactivation to show the clonal nature of cancer (Linder and Gartler, 1965). Recent approaches leverage more abundant and variable genomic marks. Rare mutations, such as microsatellites and single nucleotide somatic variants, arise during development, and ensure that each cell within an individual has a slightly different genome (Fig. 1B). Relatedness between cells from the same individual can be determined by sequencing and comparing these mutations (Behjati et al., 2014; Brody et al., 2018; Carlson et al., 2012; Frumkin et al., 2005; Lodato et al., 2015; Salipante and Horwitz, 2006). However, these mutations are rare and scattered across the genome, necessitating genome-wide recovery approaches, and may not be ideal for many biological questions (Woodworth et al., 2017). Two recent studies used such an approach to quantify the number and dynamics of hematopoietic stem cells (HSCs) within single patients, taking advantage of the ability of HSCs to expand in culture (Lee-Six et al., 2018; Osorio et al., 2018). Recent cost reductions in genome sequencing and advances in variant calling allow these approaches to be scaled to the whole genomes of individual cells (Lodato et al., 2015). However, the unbiased organism-wide profiling of single-cell whole genomes is currently impractical. In the future, the signal from these somatic mutations could be combined with orthogonal lineage information from mitochondrial ATAC-seq reads or DNA methylation marks (Ludwig et al., 2019; Walther and Alison, 2016; Xu et al., 2019; Salas et al., 2018).

An alternative approach is to engineer ‘lineage recorders’ into the genome. Reporter systems, for example, have long been used to express a fluorescent marker in specific cell types (Fig. 1C). The fluorescent marker can be permanently activated by a recombinase or triggered by an external stimulus, such as heat shock or a drug. The marker is then passed on to descendant cells, and tissues or individual cells can later be examined for fluorescence, thereby allowing lineages to be traced. Moreover, the combinatorial expression of fluorescent proteins in systems such as Brainbow can label, and therefore trace, a larger number of clonal populations (Hadjieconomou et al., 2011; Livet et al., 2007; Pan et al., 2013). Cellular barcoding approaches, by contrast, employ a diverse pool of DNA tags (‘barcodes’; see Glossary, Box 1) to mark individual cell lineages (Fig. 1D) (Kebschull and Zador, 2018). These approaches overcome some of the limitations imposed by somatic mutation-tracing methods by vastly reducing the amount of material that must be recovered from sequencing of single cells. In addition, these static marks are retained by progeny and can be recovered from terminal cells. However, most cellular barcoding techniques are limited by the requirement that the DNA barcodes must be efficiently inserted into the genome of a population of cells. Owing to these integration constraints, cellular barcoding is currently limited to *ex vivo* studies, transplanted cells, viral integration or zygote microinjection. Additionally, it can be challenging to infer a branching lineage using either fluorescent reporters or cellular barcoding.

Box 1. Glossary **Barcode.** A compact DNA sequence used to record lineage or other information, often composed of multiple target sites.

**Dynamic lineage tracing.** Recording lineage information using barcodes integrated in the genome, often via genome editing or recombinase activity.

**Edits/editing.** The error-prone repair of a CRISPR-Cas9 double-stranded break at a target site, often resulting in insertions or deletions of DNA.

**GESTALT.** Genome editing of synthetic target arrays for lineage tracing (McKenna et al., 2016).

**LINNAEUS.** Lineage tracing by nuclease-activated editing of ubiquitous sequences (Spanjaard et al., 2018).

**Polylox.** An artificial DNA recombination locus based on the Cre-*loxP* system (Pei et al., 2017).

**scGESTALT.** Single cell RNA sequencing with GESTALT (Raj et al., 2018).

**ScarTrace.** Genome editing of engineered GFP targets coupled to recovery of single cell transcriptomes (Alemany et al., 2018; Junker et al., 2017).

A more recently developed approach, which we term ‘dynamic lineage tracing’ (see Glossary, Box 1), aims to diversify barcode sequences in cells during development, allowing the generation of branching lineage trees (Fig. 1E). These approaches can generate thousands of evolving sequences that can be related to each other by shared mutations (Alemany et al., 2018; Frieda et al., 2017; Junker et al., 2017 preprint; McKenna et al., 2016; Pei et al., 2017; Peikon et al., 2014; Spanjaard et al., 2018). For example, CRISPR-Cas9 can be directed to make double-stranded breaks in the genome. These breaks are then repaired by cellular machinery, sometimes in an error-prone way, resulting in insertions or deletions at the target locus. The resulting random ‘edits’ (see Glossary, Box 1) can differentiate one cell's copy of the CRISPR target sequence from another. Although the length and composition of these repairs is generally stochastic, some sequences generate a greater variation of repair sequences than others (Allen et al., 2018; Chen et al., 2018 preprint; Shen et al., 2018). A number of these CRISPR targets can then be grouped together as a barcode in a small locus in the genome, or scattered across many loci across the genome. These dynamic barcodes are also compact, and can be coupled with emerging single-cell techniques to simultaneously capture cell transcriptional state. A related strategy uses recombinases to generate barcode diversity (Pei et al., 2017; Peikon et al., 2014). In this approach, recombinase enzymes invert or excise a fragment of DNA flanked by two recognition sites. A huge diversity of DNA sequence combinations can thus be generated using this simple shuffling procedure and a large number of recognition sites closely packed into a barcode. Generally, only one locus is used in these recombinase strategies to avoid the excision of large stretches of DNA through engineered chromosome loss (Lewandoski and Martin, 1997). Notably, both CRISPR and recombinase-based dynamic lineage tracing approaches are prospective in nature; the recording construct has to be integrated into the genome of the organism, precluding their use in humans for ethical reasons.

All of the lineage tracing methods mentioned above have advantages, drawbacks and applications in modern developmental biology. In this Review, we focus on dynamic lineage tracing, which can be used to record deep branching lineage relationships. As with most new technologies, dynamic lineage tracing must overcome many hurdles to move past the demonstration phase to widespread acceptance and utility. Here, we discuss the experimental design, implementation and analytical challenges associated with dynamic lineage tracing, as well as its application to open questions in biology.

## Barcode selection and integration

Dynamic lineage tracing approaches record information at a predefined location in the genome, commonly referred to as a barcode. Barcodes contain one or more DNA elements that can be manipulated to record information. For example, CRISPR lineage-tracing barcodes can have one or more target sites within each barcode, while recombinase barcodes can have multiple pairs of recognition sites, all in a relatively short barcode segment. The number of barcodes and their mode of insertion into the genome have significant implications for both the downstream analysis and the ability to generate and maintain the respective genetically engineered animals. Single integration approaches can take advantage of identified ‘safe harbor’ locations in the genome, where barcodes can be integrated without perturbing cellular function, such as the ROSA26 locus in mice and phiC31 in flies (Bischof et al., 2007; Sadelain et al., 2011). Other strategies use multiple genomic barcodes integrated semi-randomly throughout the genome, generally with transposon or viral integration strategies. These multiplexed techniques have, in aggregate, a greater storage capacity but also require the recovery of multiple genomic targets (discussed below). In addition, as the different chromosomes carrying these integrations will segregate in progeny, multiple barcode approaches require breeding strategies that retain sufficient integrations in an animal line (Kalhor et al., 2018; Chan et al., 2018). Random barcode integration can also disrupt existing genes, leading to unintended phenotypes; indeed, it is known that lentiviral integration is biased towards open chromatin, i.e. active stretches of DNA (Demeulemeester et al., 2015). An alternative approach is to engineer all aspects of the recording construct into an artificial chromosome system (Oshimura et al., 2015). Such a system would allow all recording components to be integrated in a single step, and would be compatible with a number of mouse models. However, it is unclear how well these synthetic chromosomes would be retained by cells when they are repeatedly targeted for double-stranded breaks by enzymes such as CRISPR, or whether barcode diversity rates would be similar to those seen with endogenous chromosomes.

Once the barcode or barcodes are encoded in the genome, the next step is to activate the recording of lineage information. This usually entails delivering the recombinase or CRISPR system into cells to trigger barcode modification. To begin recording from the single cell zygote, CRISPR components, such as Cas9 mRNA or protein, and/or guide RNAs, can be injected directly into the founding zygote or egg (Alemany et al., 2018; Junker et al., 2017 preprint; McKenna et al., 2016; Spanjaard et al., 2018). Recording can begin almost immediately in all embryonic cells, but eventually the injected CRISPR components are diluted or degraded, constraining lineage recording to the early stages of development. A parallel approach has used mismatched guide RNAs to slow the rate of editing to better mirror the slower cell cycles observed in the early mouse embryo (Chan et al., 2018). Alternatively one can drive expression of a critical component such as the recombinase or the Cas9 enzyme using ubiquitous promoters integrated into the genome (Chan et al., 2018; Kalhor et al., 2018; Pei et al., 2017). However, these approaches often require additional breeding, line generation and assessment of the accuracy and recording efficiency. A more-targeted lineage recording approach can be taken when the biological question is more focused. For example, promoters that are active in specific cell types can be used to drive recording, restricting lineage recording to a particular subpopulation. Alternatively, environmental stimuli can be used to drive expression by coupling recorder components to drug- or heat-inducible promoters (Das et al., 2016; Raj et al., 2018). Such recorder constructs can be activated at various time points, providing a real-time dimension to lineage recordings (Raj et al., 2018). The two approaches can also be combined, with subsets of recording constructs being active during early development while others record later cell divisions.

## Barcode recovery

**Fig. 2.**
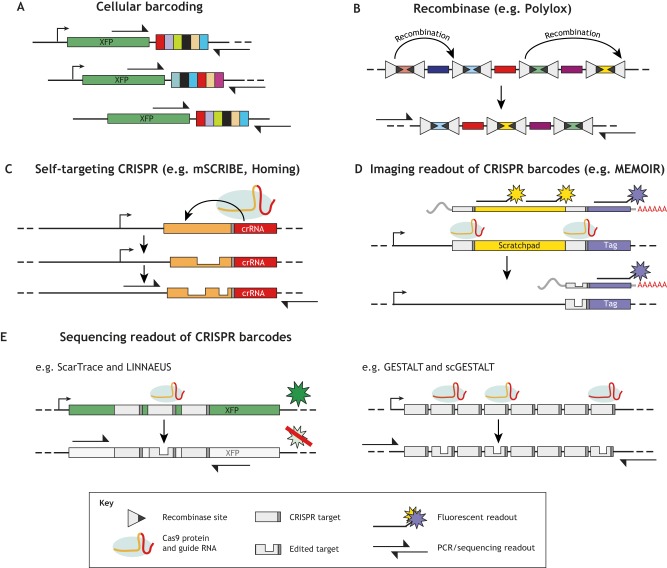
**DNA barcoding and recovery using prospective lineage recording.** (A) Cellular barcoding introduces a diverse population of nucleotide tags into the genome, typically using viral integration or transposition. These static barcodes can be read by PCR and short-read sequencing. (B) Barcode diversity can also be generated with recombinases, whereby an array of DNA-binding sites can be scrambled when the recombinase is expressed. These barcodes are typically read with long-read sequencing due to their large size (see Peikon et al., 2014). (C) Self-targeting CRISPR guide RNAs target Cas9 editing to their own genomic sequence, resulting in repeated rounds of mutation that can generate barcode diversity. The resulting sequences can be read using short-read sequencing. (D) With MEMOIR, genetic regions termed ‘scratchpads’ are inserted into the genome and collapsed by CRISPR editing. The resulting scratchpads are expressed as mRNA and detected with fluorescent *in situ* hybridization. (E) Alternatively, multi-target barcodes – in one or more locations in the genome – can be edited with a single guide RNA or distinct guide RNAs to incrementally add information to the barcode during development. There barcodes are typically recovered using short-read sequencing.

In all dynamic lineage-tracing strategies, the lineage barcodes must be recovered from the cells of interest, i.e. the information collectively encoded in the barcode(s) must be captured and assigned to each individual cell (Fig. 2). There are two major recovery strategies in dynamic lineage-tracing systems: DNA sequencing and fluorescent probe hybridization. The choice of which recovery strategy to use is linked to the initial design of the recording construct.

Short-read sequencing is used for both static cell barcoding and CRISPR-based approaches that have a smaller DNA footprint (Fig. 2A,C,E) (Alemany et al., 2018; Junker et al., 2017 preprint; McKenna et al., 2016; Raj et al., 2018; Spanjaard et al., 2018). This approach was used in the first generation of dynamic lineage-tracing approaches, although they were limited in their resolution: lineage information was recovered from bulk DNA, precluding single cell approaches. These studies focused instead on entire embryos, dissected organs or sorted cell populations. The expression of lineage recordings as messenger RNA (mRNA) can allow capture alongside the transcriptome via single cell RNA sequencing. This technical advance was recently achieved using related approaches in zebrafish and mouse embryos (Alemany et al., 2018; Chan et al., 2018; Raj et al., 2018; Spanjaard et al., 2018). While each approach differed with regard to the details of the barcode and the mode of CRISPR delivery, they all captured the transcriptome and lineage recordings simultaneously from single cells using variants of single cell RNA sequencing. However, it is already clear that capturing the lineage barcode from all cells will be a major technical constraint; the methods published so far fail to capture barcodes from every cell. For example in the study by Raj et al., barcode transcripts were recovered from only 6-28% of zebrafish cells. This loss is potentially due to some combination of: (1) insufficient transcription, either in a cell type-specific manner or from transcriptional bursting; (2) poor RNA stability, polyadenylation and/or capture during library preparation; (3) the stochastic nature of amplification in single cell RNA sequencing (Shapiro et al., 2013); or (4) loss of the PCR primer handles from long deletions (Kosicki et al., 2018). Furthermore, even when a barcode is captured, intersite deletions may erase lineage information (McKenna et al., 2016; Salvador-Martínez et al., 2019). Simulations have shown that this can impact the accuracy of downstream tree reconstruction, having a larger effect as the number of target sequences increases within the barcode (Salvador-Martínez et al., 2019). Increasing the number of barcodes, each with fewer targets, lowers the chance of intersite deletions and increases the chance of partial barcode recovery, but also limits complete capture of all barcodes (Alemany et al., 2018; Junker et al., 2017 preprint; Spanjaard et al., 2018). It is possible that higher expression or stabilization of RNA species could increase capture (Horstick et al., 2015; Rabani et al., 2017), or perhaps new advances in single cell-based approaches will be required.

Recombinase approaches generally require longer DNA constructs, as pairs of recombinase sites and their intervening sequence are longer than CRISPR targets. As such, these barcodes must be recovered using long-read sequencing technologies such as PacBio sequencing (Fig. 2B) (Pei et al., 2017; Peikon et al., 2014). This currently limits their integration with single cell methods and limits the number of barcodes that can be recovered. However, the advantage of these recombinase-based recording constructs is that they may provide predictable hybridization sequences for compatibility with existing *in situ* sequencing approaches (Frieda et al., 2017). Moreover, the inversion or deletions generated using recombinases resolve without creating double-stranded breaks, avoiding large unintended deletions, which can confound recovery in CRISPR systems (Kosicki et al., 2018).

Fluorescent probe hybridization has been adapted to recover barcodes *in situ* using a visual readout. For example, an approach termed MEMOIR (memory by engineered mutagenesis with optical *in situ* readout) leverages single molecule RNA fluorescence hybridization to recover multiple lineage barcodes from intact cells (Frieda et al., 2017). MEMOIR barcodes are expressed as mRNA and are composed of two parts – a unique tag that identifies each integration and a shared set of ten identical CRISPR targets called a scratchpad (Fig. 2D). When Cas9 and the guide RNA are expressed, large deletions collapse these scratchpads stochastically over time. Barcode recovery involves hybridizing to the tag sequence to identify integrations, and hybridization to the common scratchpad to determine whether it is intact or deleted. Recovery via this approach is exceptional; 90% barcode signal recovery is observed in embryonic stem cells (Frieda et al., 2017). The challenges are now to intersect this method with transcriptome recovery and to automate barcode recovery for hundreds of thousands of cells. Regardless of the approach – sequencing or imaging – the challenge of large-scale lineage recovery will be an important consideration in future studies.

## Lineage tree construction

The next step is to infer relationships between the recovered barcodes and build a lineage tree. This task has enormous computational challenges [reviewed by Tritschler et al., 2019 (also in this issue)]. For *n* sampled cells, there is a super-exponential number of ways to build a tree: over 34 million different rooted trees can be made from only 10 terminal cells (Felsenstein, 1978). The first step is to define a distance metric that scores relatedness between barcoded cells. This can be challenging, given the incomplete and noisy nature of the dynamic lineage-tracing barcodes, with issues such as incomplete capture, sequence alignment problems and intersite deletions in CRISPR approaches, or barcode excision in recombinase strategies. Some methods, such as the recombinase strategy Polylox (see Glossary, Box 1), enumerate all possible barcodes and assign a probability of the unaltered barcode recombining to each terminal state (Pei et al., 2017); such an approach could also be adapted to describe the distance between recombined barcodes. CRISPR approaches tend to compare the identity of editing events shared between two barcodes, treating each insertion or deletion as an independent event (Alemany et al., 2018; Chan et al., 2018; McKenna et al., 2016).

The next task is to convert these barcode relationships into a tree of cell divisions. Custom algorithms have been developed to achieve this based on maximum likelihood (Chan et al., 2018) or network graphs (Spanjaard et al., 2018). Others have adapted methods from the field of phylogenetics (see Box 2), which has a long history of addressing conceptually similar problems and has developed a number of techniques and approximations to tackle the complexity of tree construction (St. John, 2017). However, the data collected by these new lineage-tracing techniques upend several assumptions that make phylogenetics more tractable, and care should be taken when adapting these methods (see Box 2; Feng et al., 2019 preprint).

Box 2. From phylogenetics to lineage reconstruction Many lineage-tracing tools borrow techniques and terminology from phylogenetics – the study of evolutionary history and relationships between biological entities. However, it is important to be aware of the assumptions made by phylogenetic models when adapting them for lineage tracing (Yang and Rannala, 2012). Phylogenetic studies based on nucleotide changes typically examine a limited number of species and appreciably more sites (i.e. nucleotides in a gene or genome). These data together represent a wide and short matrix (see Figure, top). Dynamic lineage tracing, by contrast, captures thousands of cells and their corresponding barcode sequences. These barcodes are made up of a limited number of mutable sites, leading to a tall and narrow matrix (see Figure, bottom). These differences make it difficult to directly apply phylogenetic methods designed for wide and short matrices to lineage-tracing data.

Dynamic lineage-tracing data also break several assumptions beyond these structural differences. First, CRISPR target sites can have highly variable mutability rates, with some sites highly mutated and others completely intact (1 in Figure). Mutations are also ‘locked in’ such that deletions or insertions prevent further recording. Phylogenetic models such as the general time reversible (GTR) model assume that nucleotide substitutions are reversible. Some phylogenetic models also assume that substitution rates are identical or very similar, and all models need at least a rate estimate of nucleotide substitutions. However, DNA repair can favor certain repair outcomes over others, and this can change across organisms or developmental timepoints (Haeussler et al., 2016). Second, large deletions can destroy PCR primer sites that flank the barcode, leading to biased dropout of data from subpopulations of cells (2 in Figure). Third, in multi-site barcodes, large deletions between two sites may erase previous mutations (striped deletion, 3 in Figure). In both single- and multiple-target barcodes, there is also a chance that a target is edited again, destroying a previous signal. Fourth, independence between mutation sites is a core assumption for many phylogenetic methods, which is violated in many CRISPR lineage-tracing systems where large deletions can ablate numerous targets within a single event (4 in Figure). These unique challenges present an opportunity for new tree building techniques, such as maximum likelihood models where branch lengths can indicate developmental time, and for computational strategies that integrate the resulting trees with other single cell data.

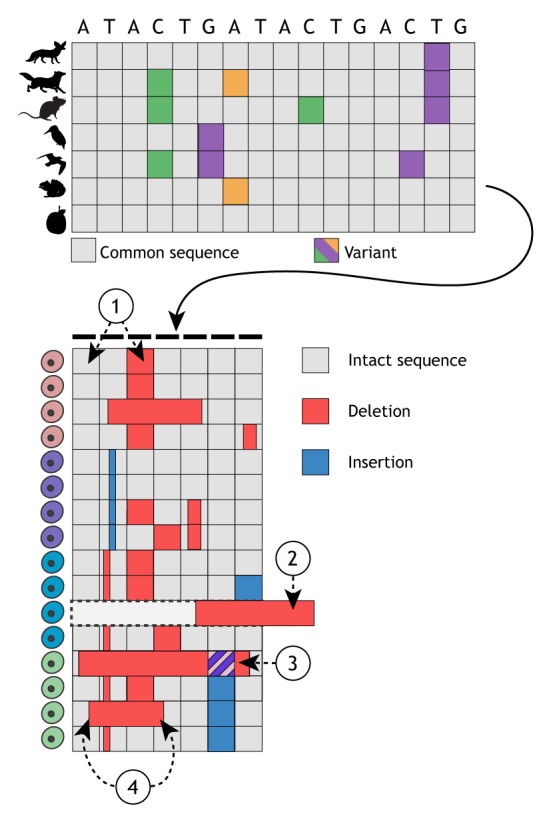

A common reconstruction technique borrowed from phylogenetics is maximum parsimony, which minimizes the number of changes (mutations or edits) over the tree as a whole. As the resulting branch lengths are equal to the number of mutations, this algorithm can simplify tree construction. However, it offers little information about the true developmental time between cell divisions. Care must also be taken to ensure that the underlying model assumptions are considered. For example, certain phylogenetic models, such as Camin-Sokal parsimony, assume an unaltered starting barcode and an irreversible transition from the unedited to the edited targets. When making comparisons with new emerging methods, it is important to consider these assumptions (Spanjaard et al., 2018). Other challenges include homoplasy, where identical marks are acquired independently on different branches of a tree. This is relevant for both CRISPR-Cas9 methods, where certain sequences produce stereotypical outcomes (Allen et al., 2018; Chen et al., 2018 preprint; Frieda et al., 2017; Shen et al., 2018; van Overbeek et al., 2016), and for recombinase methods, where there are a low number of potential outcomes (Pei et al., 2017; Peikon et al., 2014). If not dealt with, homoplasy can lead to incorrect relationships being assigned to unrelated cells. The field of tumor phylogenetics has already adapted many of these methods for the analysis of tumor evolution, and the reuse of strategies developed therein could benefit the lineage reconstruction field (Schwartz and Schäffer, 2017).

Last, the trees generated by the composite experimental and computational methods described above must be validated against known biology. Dynamic lineage tracing has recovered known cell/lineage relationships during germ layer patterning (McKenna et al., 2016), in regions of the brain (Raj et al., 2018) and within progenitors in the hematopoietic system (Alemany et al., 2018); but what resolution can we expect from these new lineage-tracing trees? Simulations offer insight (Frieda et al., 2017; McKenna et al., 2016; Salvador-Martínez et al., 2019), but experimental validation will also be important. Future benchmarking efforts that combine and contrast multiple lineage-tracing methods in well-understood biological systems, or in model organisms such as *C. elegans* that have a known lineage, will provide the most comprehensive assessment (Packer et al., 2019 preprint).

## Biological insights obtained from recent single cell dynamic lineage-tracing studies

The new tools discussed above have the potential to transform our understanding of tissue and organ development, both in terms of embryogenesis and homeostasis, and in the context of regeneration and disease. Below, we focus on two broad classes of biological questions that can be addressed with single cell lineage tracing: building the animal and maintaining the adult. First, we discuss how combining lineage tracing with single cell sequencing can allow historical annotation of embryonic cell fate specification and commitment. Second, we highlight how these approaches could resolve controversies over the differentiation potential of stem and progenitor cells within normal and regenerating tissues.

### Building the animal: cell types and fate specification

When and where are cell types specified in the developing embryo? Decades of embryological observation and manipulation have given us a rough draft, but dynamic lineage tracing now offers single cell granularity of the molecular processes that govern differentiation. With the advent of massively parallel methods for single cell RNA sequencing of tissues, organs and whole animals, we will soon know the complete parts list of cells for constructing complex animals. A challenge for interpreting these atlases, however, is to understand how and where cells are made, and how they interact to form complex systems.

Three strategies have been used to infer paths of cellular differentiation from single cell atlases. First, when the sample contains both the progenitor and differentiated cell types, it is often possible to construct trajectories that describe cell-type differentiation (Bendall et al., 2014; Shin et al., 2015; Trapnell et al., 2014; see Tritschler et al., 2019 in this issue). Second, single-cell atlases of multiple time points can be stitched together into developmental trajectories that describe cell fate acquisition across developing embryos (Briggs et al., 2018; Cao et al., 2019; Farrell et al., 2018; Pijuan-Sala et al., 2019; Wagner et al., 2018). Third, the integration of atlases with molecular imaging or tissue sectioning [reviewed by Mayr et al., 2019 (in this issue)] allows inference of the spatial organization of cells within tissues and embryos (Achim et al., 2015; Junker et al., 2014; Karaiskos et al., 2017; Satija et al., 2015; Ståhl et al., 2016).

Dynamic lineage tracing can serve as a parallel and complementary approach to trajectory inference by providing a ground truth framework for interpreting atlases. For example, single cell lineage tracing can identify cells with the same transcriptional state but with divergent origins. This is particularly important when studying cells of similar identity in spatially discrete regions of the embryo (e.g. eye development or somitogenesis) or for dissecting the contributions of individual cells within a population of a given cell type (e.g. HSCs or other stem cells). As an example, an approach called Tracer-seq used cellular barcoding in zebrafish to trace the contribution of embryonic cells to various cell types (Wagner et al., 2018). When overlaid onto a developmental trajectory of zebrafish embryogenesis, these barcodes traced explicit lineage relationships between cells. A similar strategy has been used to trace hematopoiesis in mice (Weinreb et al., 2018 preprint).

Dynamic lineage tracing can also record cell divisions after the tags have been introduced, thereby delineating the relationships between descendants (Fig. 1D). For example, CRISPR lineage tracing has been implemented in zebrafish and worms (Junker et al., 2017 preprint; McKenna et al., 2016; Schmidt et al., 2017), and more recently in mice (Chan et al., 2018; Kalhor et al., 2018), to capture early regional specification events during embryogenesis. This includes the initial segregation of multipotent cells into mesoderm, endoderm and ectoderm, and the diversification of these germ layers into more specialized precursor cells. These approaches generally sampled whole organs from adult animals. Barcodes found within organs and tissues clustered more closely with others from the same germ layer instead of across germ layers. These barcodes also allowed tracing of the entire zebrafish hematopoietic system back to a handful of pre-gastrulation embryonic cells (Junker et al., 2017 preprint; McKenna et al., 2016).

These approaches can also be used in more targeted studies. One such application labeled embryonic progenitors in the zebrafish tail bud, providing insights to the cell fate decision between neural and mesodermal fates (Attardi et al., 2018). Another study applied CRISPR lineage tracing to discover how the spatial coordinates of the mouse brain are established, determining that the anterior-posterior axis is formed earlier in development than that of the left-right axis (Kalhor et al., 2018). Finally, these approaches can be combined with classic genetic and cell transplantation techniques. For example, genetically mutant zebrafish cells labeled with CRISPR-edited barcodes have been transplanted into host embryos to rule out a cell-autonomous requirement for the transcription factor Nanog during germ layer specification (Gagnon et al., 2018).

The intersection of CRISPR lineage tracing and single cell RNA sequencing has further improved the resolution of these studies. In an approach termed scGESTALT (see Glossary, Box 1), the lineage relationships within a single cell atlas of the juvenile zebrafish brain were defined (Raj et al., 2018). Using editing at both embryonic and larval stages, it was shown that progenitors remain spatially restricted to distinct regions of the growing brain, with minimal cell movement between these regions. Furthermore, the generation of lineage trees that relate different cell types within in the brain uncovered a novel lineage branchpoint that generates distinct cell types in the hypothalamus. In a similar vein, the LINNAEUS technique (see Glossary, Box 1) was used to perform embryonic editing in zebrafish to study the relationships between cell types in various organs (Spanjaard et al., 2018). Specifically, this method was used to reveal lineage hierarchies that describe aspects of organogenesis in the heart, liver and telencephalon. Moreover, the combination of lineage and cell type information in this context allowed the authors to define distinct lineages that contribute to the endocardium and myocardium in the heart. In another recent study, embryonic CRISPR editing and single cell transcriptional state profiling was used to define the split of embryonic neuroectoderm into the left and right aspects of the brain and eyes, providing an example of how these tools can provide spatial information missing from developmental trajectories alone (Alemany et al., 2018).

Lineage tracing can also uncover convergent events in developmental lineages, where distinct lineage paths lead to a shared cell type. For example, delays and detours along developmental trajectories were recently identified within single-cell atlases of zebrafish and mouse embryogenesis (Cao et al., 2019; Farrell et al., 2018; Pijuan-Sala et al., 2019; Wagner et al., 2018). Cells in several developmental trajectories (axial mesoderm, neural plate/lateral plate, endoderm and others) appeared to differentiate based on their molecular identity but later converged into a common transcriptional state. Convergence of lineages to a common cell type is also seen in the heart: cardiomyocytes have embryonic origins in both the mesoderm and neural crest (Keyte and Hutson, 2012), with consequences for adult heart function (Abdul-Wajid et al., 2018). The application of CRISPR lineage tracing in mouse embryos has also described the convergence of discrete embryonic and extra-embryonic lineages into a common endodermal cell type (Chan et al., 2018). Although these studies have uncovered new and exciting insights into developmental trajectories, they also raise a number of questions that pave the way for future studies. For example, how common are these convergences in differentiation? Are the resulting cells equipotent and, if so, what are the implications for the formation, maintenance and regeneration of healthy tissues?

### Maintaining the adult: stem and progenitor cell potential

Lineage tracing can also help us understand the developmental potential of cells within adult organs, a topic of great interest in recent years. For practical reasons, hematopoiesis has served as a testbed for technological advances in clone tracing and cellular barcoding (Yamamoto et al., 2018). Indeed, studies of hematopoiesis established the paradigm of dedicated multipotent stem cells at the top of a differentiation hierarchy. The transplantation of barcoded HSCs back into host animals uncovered clonal dynamics between transplanted cells, highlighting variable contributions to different branches of the blood lineage tree and a change in the composition of the bone marrow over time (Carrelha et al., 2018; Lu et al., 2011; Naik et al., 2013). However, these transplanted cells may not recapitulate native stem cell activity. A creative solution to this problem is to use random transposon insertions to uniquely mark endogenous HSCs in an animal. Such an approach has been used to trace population dynamics and cell contributions during native hematopoiesis in the mouse, defining population clonal dynamics over time and discrete lineage contributions between HSCs (Rodriguez-Fraticelli et al., 2018; Sun et al., 2014). In a parallel approach, the Polylox system was applied to mouse hematopoiesis to delineate lineage hierarchies of blood development (Pei et al., 2017).

Dynamic lineage tracing provides a complementary approach for understanding lineage hierarchies in hematopoiesis and in other stem and progenitor populations. For example, CRISPR-based lineage tracing has been applied to understand the lineage connections between progenitor and differentiated cells in zebrafish: tracing clones of embryonic cells using GESTALT (see Glossary, Box 1) suggested that clonal dynamics might drive population drift within all adult tissues and organs, not just blood (McKenna et al., 2016). More recently, scGESTALT has been applied to the developing brain to trace the continuous production of differentiated cells from pools of neural and oligodendrocyte precursors (Raj et al., 2018). Another method, termed ScarTrace (see Glossary, Box 1), has been used to identify immune cells in the zebrafish fin, showing that they have an embryonic origin distinct from the rest of the haematopoietic system (Alemany et al., 2018). These approaches can be applied in virtually any tissue or organ, and in the future may clarify controversies of whether there are ‘professional’ stem cells outside of the blood (Clevers and Watt, 2018). Is it possible that other cell types moonlight as stem or progenitor cells? Although examples of multipotent stem cell potential exist in the skin, intestine, liver, lung and other tissues, this activity often cannot be directly ascribed to a dedicated stem cell, and instead is found distributed across a population of progenitor cells or emerges after injury (Merrell and Stanger, 2016). New lineage-tracing tools will allow researchers to better label cells and track their contributions to tissue homeostasis in normal and diseased states.

Single-cell dynamic lineage tracing could also be applied to studies of regeneration (Mokalled and Poss, 2018; Rajagopal and Stanger, 2016). A fundamental question of regeneration is whether there are regeneration-specific lineage programs, or whether regeneration can be seen as the recapitulation of normal organ development. In the zebrafish caudal fin, a well-studied regeneration system, a consensus has emerged to suggest that the blastema (the region of regenerating tissue) contains few if any multipotent dedifferentiated cells. However, osteoblasts retain some potential to dedifferentiate and contribute to other cell types in a regeneration-specific manner, a finding that was initially demonstrated with classic lineage tracing and recently confirmed using dynamic lineage tracing (Alemany et al., 2018; Junker et al., 2017 preprint; Stewart and Stankunas, 2012; Tornini et al., 2017; Tu and Johnson, 2011). A blastema is also formed from adult cells during limb regeneration in axolotls (Haas and Whited, 2017; Tanaka, 2016). Recently, time course single cell RNA sequencing was applied to this process to reveal cells that dedifferentiate into embryonic-like cells that exhibit multipotency during limb regeneration (Gerber et al., 2018; Leigh et al., 2018). Together with a second study using CRISPR lineage tracing (Flowers et al., 2017), these data argue against professional regenerative stem cells and instead support a model in which adult cells can dedifferentiate and proliferate during axolotl regeneration.

Overall, these studies highlight promising applications of dynamic lineage tracing to monitor the contributions of all cells during development or within a tissue during tissue homeostasis and response to injury. Initial studies are encouraging, but must be caveated by the fact that they are based on very small numbers of individuals. Remaining challenges include finding ways to scale these approaches and to develop methods to compare across individuals or species.

## Conclusions and perspectives

**Fig. 3.**
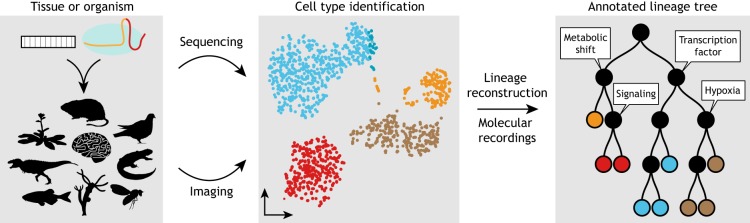
**The future of dynamic lineage tracing.** Single cell transcriptomes and barcode recordings can be recovered from a variety of animals, tissues and organoids using sequencing and/or imaging approaches. These data can be organized into cell types using dimensionality reduction approaches. The cell lineage tree serves as an organizing scaffold to relate single cells and cell types with embedded recordings of spatial context, signaling landmarks and other measures of cellular state. The output will be richly annotated trees of development and disease.

The recent explosion of single cell data sets, including whole-organism atlases, provides an unprecedented view of development. The challenge is to link these static views of cell states together into a dynamic view of how cell types arise, and how they are regulated, maintained and ultimately dysregulated in disease (Fig. 3). It is becoming clear that single cell transcriptomes alone cannot solve this problem. Lineage tracing has shown promise as a scaffold to link cell state to cell fate, and this union with single cell transcriptomics provides the potential to explore the underlying molecular mechanisms that drive cell fate decisions (Kester and van Oudenaarden, 2018; Woodworth et al., 2017). There will be opportunities to enrich this scaffold with fate choices documented by a new generation of DNA recording constructs that respond to transient endogenous events, allow time-resolved recordings, use distributed barcodes for deeper recording and provide independence from potentially dangerous DNA breaks (Biddy et al., 2018; Farzadfard et al., 2018 preprint; Halperin et al., 2018; Perli et al., 2016; Schmidt et al., 2018; Sheth et al., 2017; Shipman et al., 2016; Tang and Liu, 2018). It is also an enticing challenge to intersect prospective lineage tracing with the powerful tools of microscopy. An ideal system would recover molecular cell state and historical recordings, all in the native spatial context of an intact organism. Although it is early days, there have already been promising steps towards this goal (Chen et al., 2015; Frieda et al., 2017; Lee et al., 2014; Shah et al., 2017; Wang et al., 2018). Interesting avenues for future work are the aggregation of data from multiple recording constructs within an organism, and the aggregation of stochastic lineage choices across many individuals. This future field of statistical development will incorporate developmental variation into models of fate choices, plasticity and function in the homeostasis of adult tissues.

With all of these evolving tools, the current challenge for biologists is to choose the best technology for their question. Dynamic lineage tracing tools are still emerging, and each approach comes with strengths and weaknesses. For computational biologists, there will also be exciting opportunities to develop new tools, especially as lineage information is coupled with single-cell measurements such as RNA sequencing, ATAC sequencing and others (Kelsey et al., 2017; Stuart and Satija, 2019). The merger of these efforts will no doubt shed light on previously intractable questions in biology, and allow the characterization and engineering of fate choices in animal models and human organoids. Ultimately, a multifaceted view of development will be provided by dynamic lineage tracing, the broad capture of single cell states and high-dimensional phenotyping. These tools will provide enormous datasets for developmental biologists looking to understand one of the most complex construction projects on earth – the development of the adult human.
